# PF‐06409577 inhibits renal cyst progression by concurrently inhibiting the mTOR pathway and CFTR channel activity

**DOI:** 10.1002/2211-5463.13459

**Published:** 2022-07-04

**Authors:** Limin Su, Haoxing Yuan, Haoran Zhang, Ruoqi Wang, Kequan Fu, Long Yin, Ying Ren, Hongli Liu, Qian Fang, Junqi Wang, Dong Guo

**Affiliations:** ^1^ Jiangsu Key Laboratory of New Drug Research and Clinical Pharmacy Xuzhou Medical University China; ^2^ Department of Urology The Affiliated Hospital of Xuzhou Medical University China

**Keywords:** ADPKD, AMPK, CFTR, kidney disease, mTOR, PF‐06409577

## Abstract

Renal cyst development and expansion in autosomal dominant polycystic kidney disease (ADPKD) involves over‐proliferation of cyst‐lining epithelial cells and excessive cystic fluid secretion. While metformin effectively inhibits renal cyst growth in mouse models of ADPKD it exhibits low potency, and thus an adenosine monophosphate‐activated protein kinase (AMPK) activator with higher potency is required. Herein, we adopted a drug repurposing strategy to explore the potential of PF‐06409577, an AMPK activator for diabetic nephropathy, in cellular, *ex vivo* and *in vivo* models of ADPKD. Our results demonstrated that PF‐06409577 effectively down‐regulated mammalian target of rapamycin pathway‐mediated proliferation of cyst‐lining epithelial cells and reduced cystic fibrosis transmembrane conductance regulator‐regulated cystic fluid secretion. Overall, our data suggest that PF‐06409577 holds therapeutic potential for ADPKD treatment.

## Graphical abstract

Over‐proliferation of cyst‐lining epithelial cells and excessive cystic fluid secretion are the most important causes of renal cyst enlargement. In this study, we evaluated the therapeutic benefits of PF‐06409577, an AMPK activator, in ADPKD. PF‐06409577 effectively down‐regulated mTOR pathway‐mediated proliferation of cyst‐lining epithelial cells and CFTR‐regulated cystic fluid secretion, delaying the progression of ADPKD significantly. PF‐06409577 holds therapeutic potential for ADPKD.

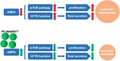

**Abbreviations**
4EBP1: 4E‐binding protein 1
8‐Br‐cAMP: 8‐bromoadenosine 3′,5′‐cyclic monophosphate
ADPKD: autosomal dominant polycystic kidney disease
AKT: AKT serine/threonine kinase
AMPK: adenosine monophosphate‐activated protein kinase
CFTR: cystic fibrosis transmembrane conductance regulator
ESRD: end‐stage renal disease
FSK: forskolin
HE: hematoxylin and eosin staining
IBMX: 3‐isobutyl‐1‐methylxanthine
mTOR: mammalian target of rapamycin
NAFLD: non‐alcoholic fatty liver disease
NASH: non‐alcoholic steatohepatitis
p70S6K: p70 ribosomal protein S6 kinase
PC1: polycystin‐1
PC2: polycystin‐2
PF‐06409577: 6‐Chloro‐5‐[4‐(1‐hydroxycyclobutyl)phenyl]‐1*H*‐indole‐3‐carboxylic acid
PI3K: phosphoinositide 3‐kinase
TSC2: tuberous sclerosis complex 2
VEGF: vascular endothelial growth factor
WT: wild‐type

Autosomal dominant polycystic kidney disease (ADPKD) is a prevalent inherited renal disease. The disease is primarily caused by mutations in one of the two genes, *PKD1* or *PKD2*, encoding polycystin‐1 (PC1) and polycystin‐2 (PC2), respectively [1, 2]. The clinical manifestations of ADPKD include inexorable formation and expansion of multiple renal cysts in bilateral kidneys, which progressively compromise renal function. Eventually, ADPKD may lead to end‐stage renal disease (ESRD) [3].

The molecular mechanisms underlying ADPKD have been delineated in several recent studies [4, 5]. A generally accepted theory is that functional loss of PC1/PC2 disrupts intracellular Ca^2+^ homeostasis, leading to cystogenesis through activating several proliferative and fluid secretion pathways [6]. Among these pathways, the mammalian target of rapamycin (mTOR) pathway is a driving force for the hyperproliferative phenotype of ADPKD cells [7], while the cystic fibrosis transmembrane conductance regulator (CFTR) mediates excessive fluid secretion into cyst lumen and promotes cyst enlargement in the kidney [8, 9].

Interestingly, the mTOR signaling pathway and CFTR function could be concurrently mediated by the adenosine monophosphate‐activated protein kinase (AMPK). This is evident from (a) AMPK indirectly inhibited mTOR kinase activity through phosphorylating tuberous sclerosis complex 2 (TSC2) and consequently suppressed protein synthesis and cell growth [10]. (b) AMPK phosphorylated and directly inhibited CFTR, reducing channel open probability and thus inhibiting transepithelial fluid secretion [11]. Therefore, an AMPK activator may have potential therapeutic effects for the treatment of ADPKD. Indeed, a previous study reported that metformin could effectively inhibit renal cyst growth in two mouse models of ADPKD [7]. However, due to its low potency, the dose regimen of metformin in the phase II clinical trial (NCT02903511) was 500 mg every 2 weeks up to 1000 mg twice a day, almost 10‐fold the amount of tolvaptan (i.e., the drug approved by the FDA for ADPKD treatment). In this regard, an AMPK activator with higher potency is preferred for better therapeutic index and patient compliance.

6‐Chloro‐5‐[4‐(1‐hydroxycyclobutyl)phenyl]‐1*H*‐indole‐3‐carboxylic acid (PF‐06409577) activates α1β1γ1 and α2β1γ1 AMPK isoforms with potencies of 7.0 and 6.8 nm, respectively [12]. This compound has been demonstrated efficacious for the treatment of diabetic nephropathy [12]. Herein, we adopted the drug repurposing strategy and tested whether PF‐06409577 slowed renal cystogenesis through inhibition of both the mTOR pathway and CFTR function in cellular, *ex vivo* and *in vivo* models of ADPKD.

## Materials and methods

### Chemicals and reagents

PF‐06409577 (#M8095, Abmole, Shanghai, China), Forskolin (FSK; #F6886, Sigma, Shanghai, China) and 8‐bromoadenosine 3′,5′‐cyclic monophosphate (8‐Br‐cAMP; #B5386, Sigma) were dissolved in 100% DMSO as 100 mm stock solutions and stored at −20 °C. DMSO (#D2650) and 3‐Isobutyl‐1‐methylxanthine (IBMX; #I5879‐1G) were from Sigma. Anti‐AMPK (1 : 1000, #2532), anti‐p‐AMPK (1 : 1000, #2535), anti‐AKT (1 : 1000, #9272), anti‐p‐AKT (1 : 1000, #9271), anti‐p70S6K (1 : 1000, #2708) and anti‐p‐p70S6K (1 : 1000, #9234) were from Cell Signaling Technology (CST, Shanghai, China). Anti‐4EBP1 (1 : 1000, #ab32024), anti‐p‐4EBP1 (1 : 1000, #ab75767), anti‐GAPDH (1 : 1000, #ab9585) and goat anti‐rabbit IgG (1 : 5000, #ab216773) were from Abcam (Shanghai, China). CFTR_Inh_‐172 (#SF9110) was from Beyotime Biotechnology (Shanghai, China). PEG300 (#HY‐Y0873) was from MCE (Shanghai, China). Tween80 (#1716) was from Biofroxx (Guangzhou, China). Other chemicals were of analytical grade and obtained from standard commercial sources.

### Animals

Animals received humane care according to the Guide for the Care and Use of Laboratory Animals prepared by the Institute of Laboratory Animal Resources and published by the National Institutes of Health (NIH Publication 86‐23, revised 1996). Animal use was reviewed and approved by the Laboratory Animal Ethics Committee of Xuzhou Medical University (L20210226467).

### MDCK cells cyst model

Type I Madin–Darby canine kidney (MDCK) cells (ATCC no. CCL‐34) were cultured in DMEM/F12 medium containing 10% fetal bovine serum, 2 mm glutamine and 1% penicillin–streptomycin at 37 °C in 5% CO_2_. MDCK cysts were generated as previously described [13, 14]. The 400–600 MDCK cells were suspended in 0.4 mL 10 × MEM medium containing 2.9 mg·mL^−1^ collagen (PureCol, Inamed Biomaterials, Fremont, CA, USA), 100 U·mL^−1^ penicillin, 100 μg·mL^−1^ streptomycin, 27 mm NaHCO_3_ and 10 mm HEPES, pH 7.4. Cell suspensions were plated onto 24‐well plates and incubated for 1.5 h. Subsequently, a 1.5 mL medium containing 10 μm FSK was added to each well in ice‐cold collagen gel solution. The MDCK culture medium was exchanged every 12 h for a duration of 12 days. To examine the inhibitory effect of PF‐06409577 on MDCK cyst growth, 1, 3 or 10 μm PF‐06409577 was added to the medium from day 5. The cysts were pictured every 2 days and the cyst diameters (10 cysts/well and 3 wells/group) were analyzed using photoshop cs6 software (New York, NY, USA).

### Embryonic kidney cyst model

Embryonic kidneys from wild‐type C57BL/6 mice were isolated at embryonic day 13.5 and were cultured as described previously [13]. Cysts were stimulated with 100 μm 8‐Br‐cAMP to induce cyst development. To determine the concentration‐dependent inhibitory effect of PF‐06409577 on cyst development, 1, 3 or 10 μm PF‐06409577 was added into the medium. Pictures of embryonic kidneys were taken using an inverted microscope (Olympus, Tokyo, Japan). The cystic index was quantified by analyzing the ratio of cyst area to the total area of the kidney section using image‐pro plus 6.0 software (Rockville, MD, USA).

### *Pkd1^flox/flox^;Ksp‐Cre* ADPKD mice

*Pkd1* ^*flox/flox*^ *;Ksp‐Cre* ADPKD mice were generated as previously described [15]. In brief, *Pkd1* ^*flox*/+^ *;Ksp‐Cre* mice were self‐crossed to generate wild‐type (WT, *Pkd1* ^+/+^ *;Ksp‐Cre*) and ADPKD (*Pkd1* ^*flox/flox*^ *;Ksp‐Cre*) mice [13, 16]. Both WT and ADPKD mice were divided into three groups, that is, the group treated with vehicle (10% DMSO, 45% saline, 40% PEG300 and 5% tween 80), the group treated with 1 mg·kg^−1^ PF‐06409577 and the group treated with 10 mg·kg^−1^ PF‐06409577. Each group contains at least five mice. Chemicals were administered once a day by subcutaneous injection on the back using a microsyringe (Shanghai Gaoge Industrial and Trading CO., LTD, Shanghai, China) from postnatal day 6 (P6) to P12. On postnatal day 12, mice were weighted and sacrificed. The bilateral kidneys were harvested to calculate the total kidney weight to body weight (KW/BW) ratio. Renal cystic formation and development were visualized by HE‐stained kidney sections.

### Western blotting

Cell and tissue lysate preparation was performed as previously described [17]. Proteins were separated by SDS/PAGE based on molecular weight and transferred to BioTrace NT nitrocellulose transfer membrane (Pall Corporation, Show Low, AZ, USA). Subsequent to blocking, the membranes were incubated with primary antibodies at 4 °C overnight. After this, the pretreated membranes were washed three times. Following a two‐hour incubation with a secondary antibody at room temperature, the membranes were stained and the protein bands were detected using a two‐color infrared laser imaging system (Odyssey Sa, LICOR, Lincoln, NE, USA). The blots were analyzed using image j software (Version 1.53c, National Institutes of Health, Bethesda, MD, USA).

### CFTR short‐circuit current measurements

Madin–Darby canine kidney cells were seeded on transwell filters at 3000 cells per well. Cells growing to a transepithelial resistance of 1000–2000 Ω were pretreated with PF‐06409577 (0, 10 or 30 nm) for 1 h before short‐circuit current measurements. Afterward the filters were installed on the Ussing chamber system. A 5 mL culture medium containing 1.2 mm CaCl_2_, 120 mm NaCl, 10 mm HEPES, 3.3 mm KH_2_PO_4_, 25 mm NaHCO_3_, 0.83 mm K_2_HPO_4_, 1.2 mm MgCl_2_ and 10 mm mannitol (for apical) or 10 mm glucose (for basolateral) was filled into the hemichambers. The MDCK cells were firstly stimulated with 10 μm FSK and 0.5 mm IBMX. After the short‐circuit current reached a plateau phase, 30 μm CFTR_Inh_‐172 was added to decrease the short‐circuit current to a lower plateau phase. The difference between the upper plateau and the lower plateau represented the transport capacity of the CFTR channel. A signal monitoring system for the Ussing Chamber (Beijing KingTech Technology, Beijing, China) was used to continuously record the short‐circuit current.

### Statistical analysis

Statistical analysis was performed using prism 7.0 (San Diego, CA, USA). All results were expressed as means ± SEM. Each experiment was performed at least three times. Student's *t* test, one‐way ANOVA or two‐way ANOVA followed by Tukey's *post hoc* analysis were performed to assess differences between the groups. *P* < 0.05 was considered statistically significant.

## Results

### PF‐06409577 reduced cyst formation and enlargement in an MDCK cell cyst model

**Fig. 1:**
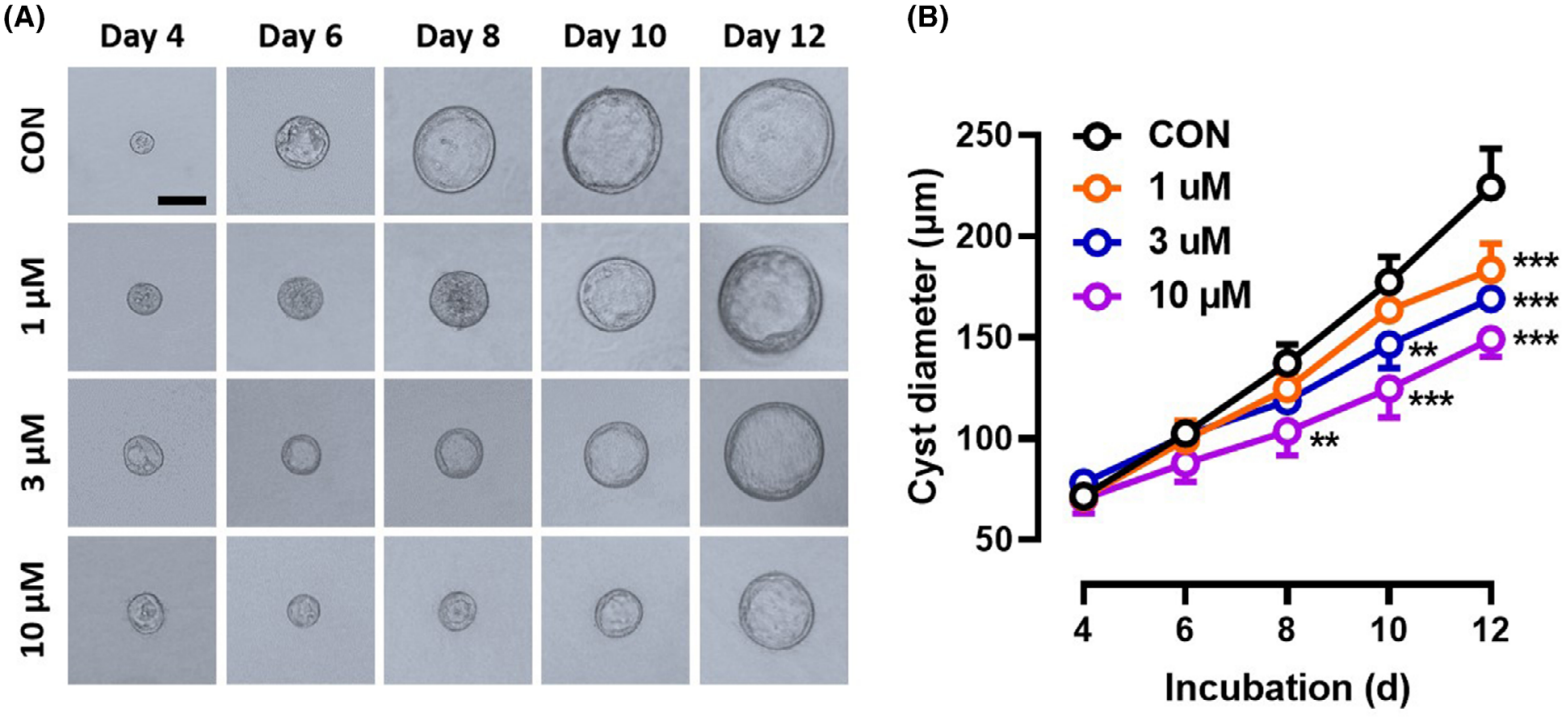
PF‐06409577 reduced cyst formation and growth in an MDCK cell cyst model. (A) Representative images of MDCK cyst growth in collagen gel in the absence or presence of 1, 3 or 10 μm PF‐06409577 from culture day 4 to day 12. Bar = 100 μm. (B) the growth curve of MDCK cysts of each group from panel (A), represented by changes in cyst diameters with the time of administration. Data are presented as means ± SEM of five independent experiments, each performed by measuring the average size of more than 30 individual cells, **P < 0.01, ***P < 0.001 vs. Control (CON), two‐way ANOVA followed by Tukey's analysis.

We first employed the MDCK cyst model, an *in vitro* model of renal cystogenesis [13], to examine the inhibitory effect of PF‐06409577 on renal cyst development and growth. Cysts started to form (diameter > 50 μm) on day 4 and continuously expanded from day 4 onward (Fig. 1A, top row). Exposure of established cysts with PF‐06409577 significantly inhibited cyst enlargement and the effect was concentration‐dependent (Fig. 1). At 10 μm, PF‐06409577 inhibited cyst growth by > 40% (Fig. 1B).

### PF‐06409577 retarded renal cyst development in an embryonic kidney cyst model

**Fig. 2:**
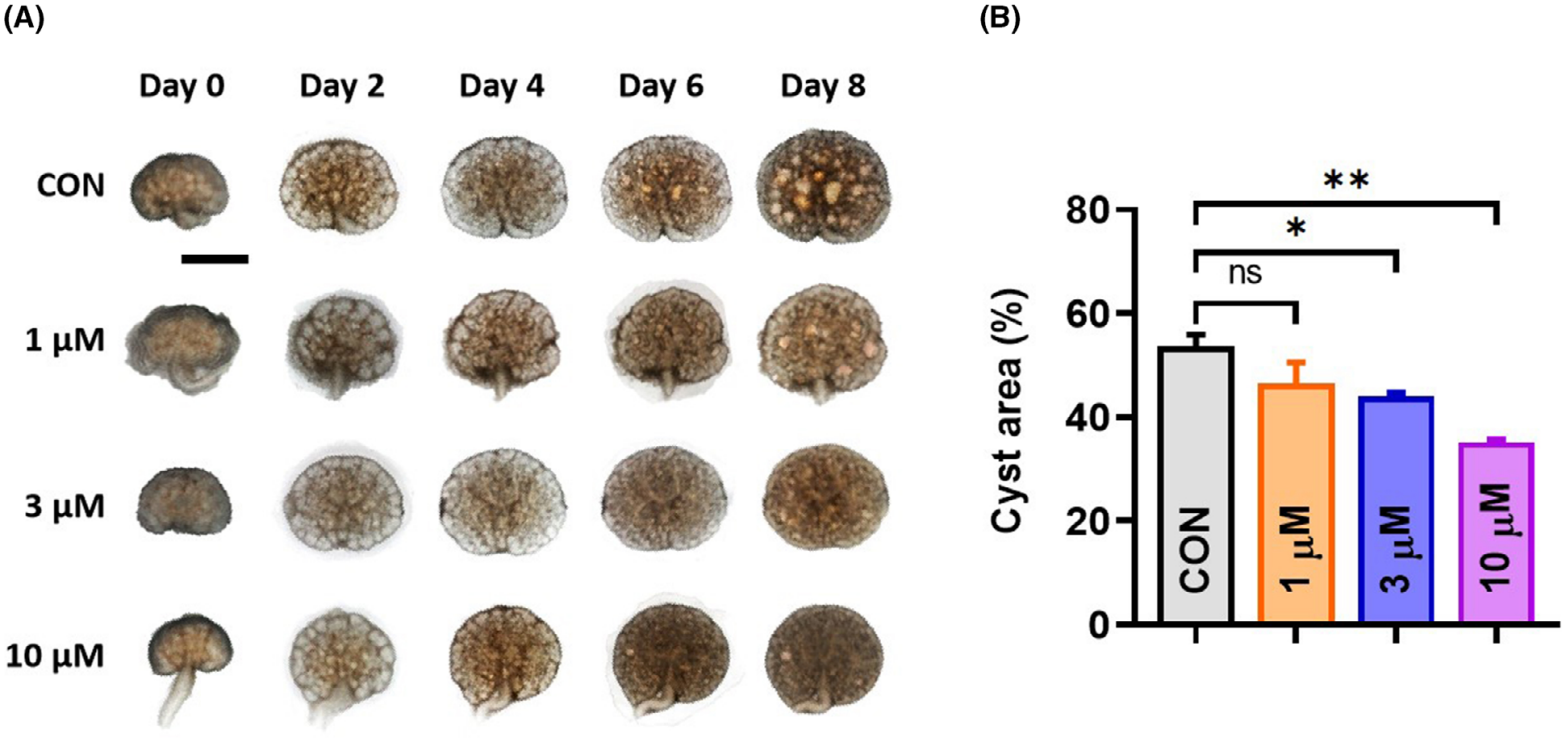
PF‐06409577 retarded cyst development and growth in an embryonic kidney cyst model. (A) Embryonic kidneys were cultured in the absence or presence of 1, 3 or 10 μm PF‐06409577 on culture days 0, 2, 4, 6 and 8. Bar = 1 mm. (B) Fractional cyst areas (%) of kidneys on culture day 8. Fractional cyst areas = (total cyst area/kidney area)*100%. Data are presented as means ± SEM. *n* = 3, ns, not significant, *P < 0.05, **P < 0.01 vs. Control (CON), one‐way ANOVA followed by Tukey's analysis.

We employed the embryonic kidney cyst model, an *ex vivo* model of renal cystogenesis, to further evaluate the inhibitory effect of PF‐06409577. In the presence of 8‐Br‐cAMP, numerous cyst structures were observed from day 2. The cysts progressively expanded over the next 6 days (Fig. 2A, top row). Upon PF‐06409577 treatment, both the number of cysts and the average size of the individual cyst were significantly reduced (Fig. 2A), as confirmed by quantitative image analysis of the fractional cyst area (Fig. 2B). As illustrated in Fig. 2B, the inhibitory effect of PF‐06409577 was concentration‐dependent, whereas 10 μm PF‐06409577 inhibited cyst growth by > 30%.

### PF‐06409577 impeded renal cyst development in *Pkd1^flox/flox^;Ksp‐Cre* ADPKD mice

**Fig. 3:**
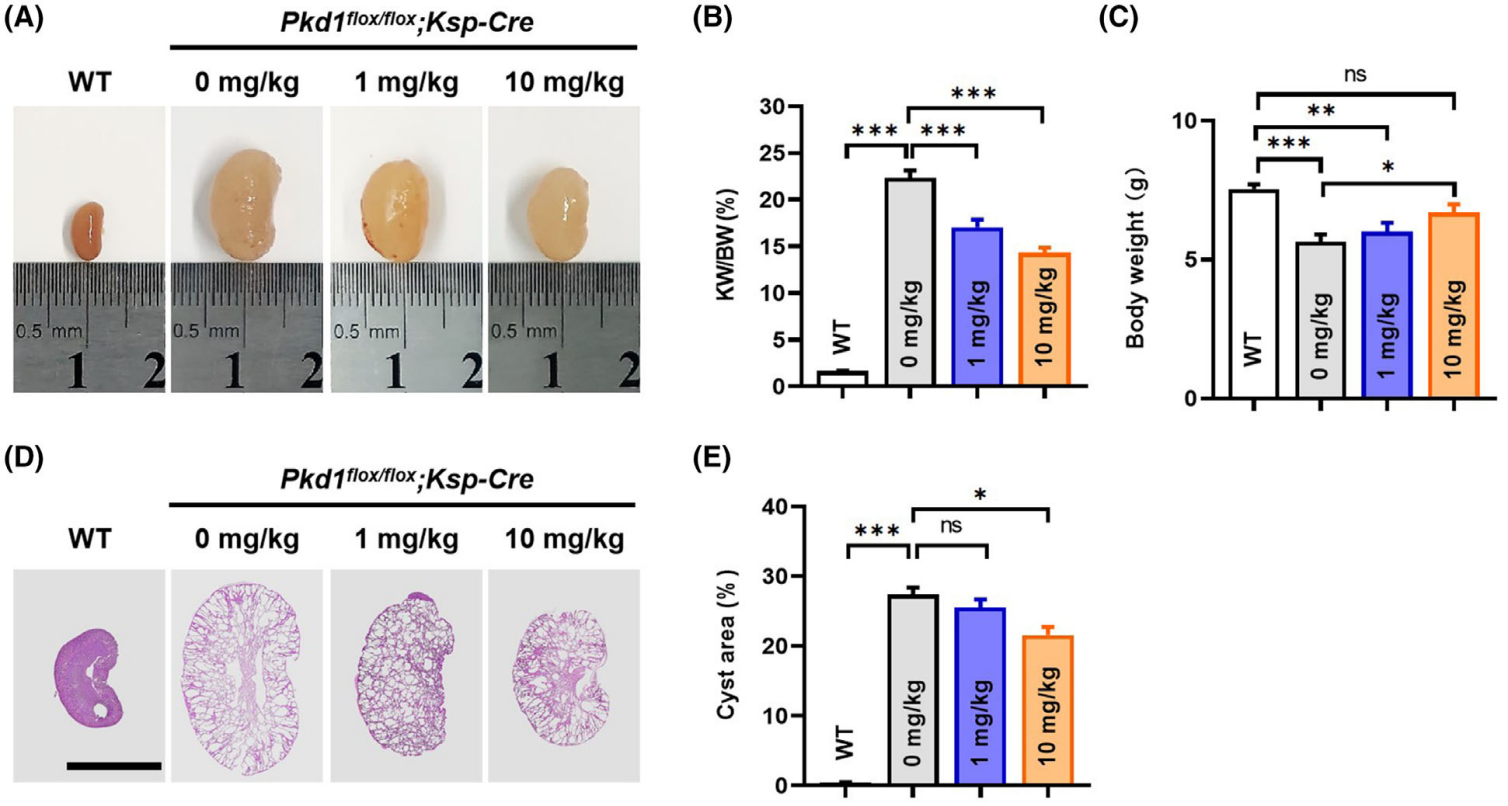
PF‐06409577 impeded renal cyst development in *Pkd1* ^*flox/flox*^ *;Ksp‐Cre* ADPKD mice. (A) Kidneys of WT (wild‐type, *Pkd1* ^+/+^ *;Ksp‐Cre*) and ADPKD (*Pkd1* ^*flox/flox*^ *;Ksp‐Cre*) mice treated with vehicle or PF‐06409577 (1 or 10 mg·kg^−1^·day^−1^) on postnatal day 12. (B) Quantification of the total kidney weight to body weight (KW/BW) ratio of each group. (C) Quantification of the body weight of each group on P12. (D) Representative HE stained images of kidneys from WT and ADPKD mice treated with vehicle or PF‐06409577. Bar = 5 mm. (E) Average cyst area (total cyst area/total kidney area) for mice of each group. Data are presented as means ± SEM. *n* = 6, *P < 0.05, **P < 0.01, ***P < 0.001, ns, not significant, one‐way ANOVA followed by Tukey's analysis.

Next, the inhibitory effect of PF‐06409577 on renal cyst progression *in vivo* was further investigated using neonatal kidney‐specific *Pkd1* knockout mice (*Pkd1* ^*flox/flox*^ *;Ksp‐Cre*) [18]. The mice were treated with PF‐06409577 (0, 1 or 10 mg·kg^−1^·day^−1^) from P6 to P12 and sacrificed on P12 to harvest the kidneys. It follows from Fig. 3A,B that kidneys from the ADPKD mice were dramatically enlarged compared to wild‐type mice (*P* < 0.0001, one‐way ANOVA followed by Tukey's analysis, *n* ≥ 5). Upon treatment with PF‐06409577, the size of the kidneys was significantly reduced. Such effect was dose‐dependent with 10 mg displaying the greatest inhibitory effect by > 35%. We also observed that ADPKD mice had a significantly decreased body weight compared with WT mice (*P* = 0.0002, one‐way ANOVA followed by Tukey's analysis, *n* ≥ 5). Upon treatment with PF‐06409577, the body weight of ADPKD mice increased significantly compared to the untreated group (*P* = 0.0287, one‐way ANOVA with Tukey's analysis, *n* ≥ 5, Fig. 3C). Further examining the HE‐stained kidney sections corroborated that the kidneys of the ADPKD mice were enlarged with multiple cysts throughout the kidney, while in PF‐06409577 treated ADPKD mice, the size of the cysts was significantly reduced with more residual renal parenchyma (Fig. 3D,E). Taken together, these results suggested that PF‐06409577 was efficacious in impeding cyst development in the kidneys of ADPKD mice.

### PF‐06409577 inhibited cell proliferation by stimulating AMPK and inhibiting the mTOR pathway

**Fig. 4:**
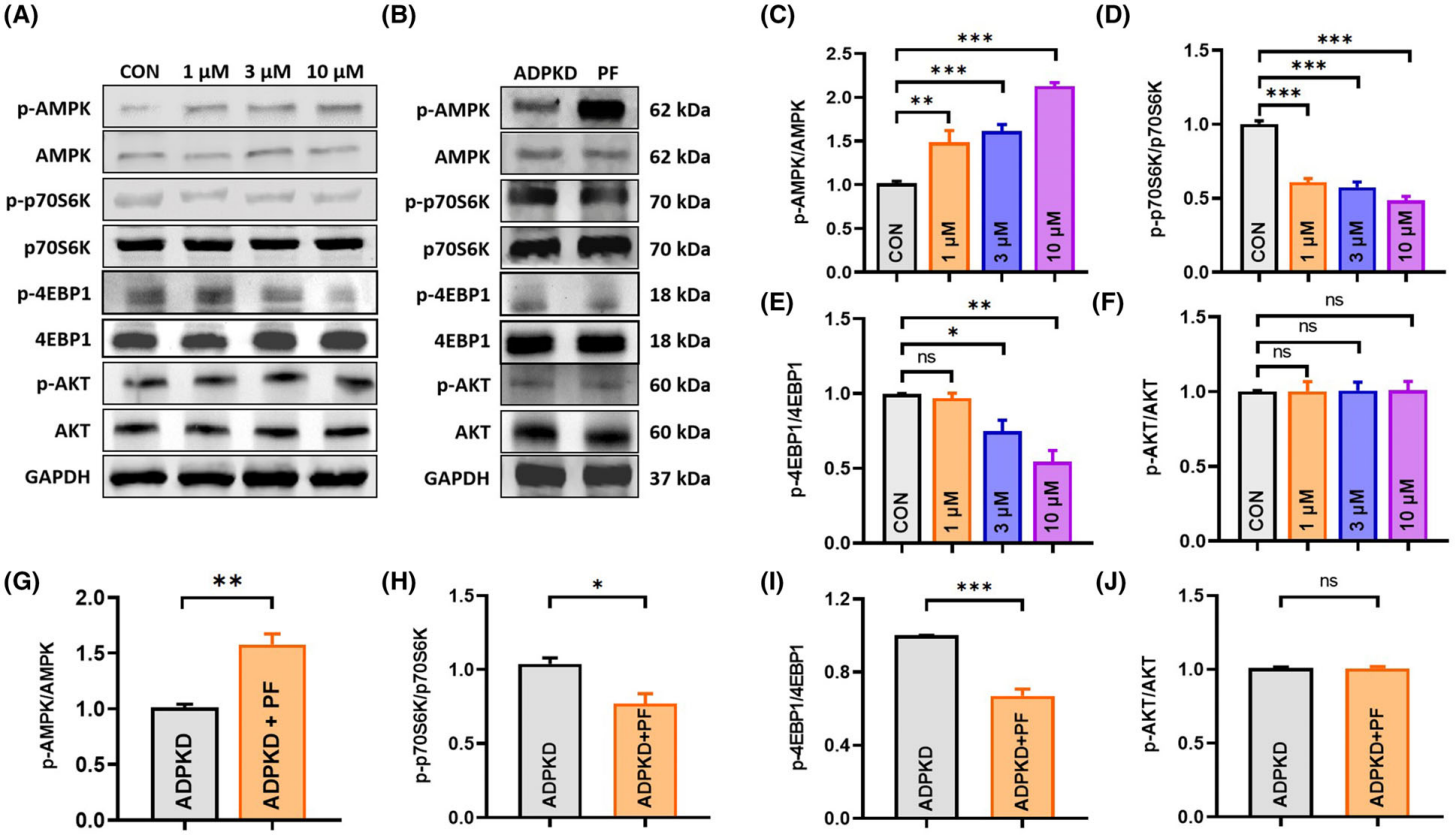
PF‐06409577 promoted AMPK phosphorylation and down‐regulated the mTOR pathway in the MDCK model and in ADPKD mouse kidneys. (A) Representative western blot of signaling proteins in MDCK cells treated with vehicle or different concentrations of PF‐06409577 (1, 3 or 10 μm). (B) Representative western blot of signaling proteins in ADPKD mouse (*Pkd1* ^*flox/flox*^ *;Ksp‐Cre*) kidneys treated with vehicle or PF‐06409577 (10 mg·kg^−1^·day^−1^), PF stands for PF‐06409577. (C) Densitometric quantification of p‐AMPK/AMPK expression. (D) Densitometric quantification of p‐p70S6K/p70S6K expression. (E) Densitometric quantification of p‐4EBP1/4EBP1 expression. (F) Densitometric quantification of p‐AKT/AKT expression. Data are presented as means ± SEM. *N* ≥ 3, *P < 0.05, **P < 0.01, ***P < 0.001 vs. Control (CON), ns, not significant, one‐way ANOVA followed by Tukey's analysis. (G) Densitometric quantification of p‐AMPK/AMPK expression in mouse kidneys from each group. (H) Densitometric quantification of p‐p70S6K/p70S6K expression in mouse kidneys from each group. (I) Densitometric quantification of p‐4EBP1/4EBP1 expression in mouse kidneys from each group. (J) Densitometric quantification of p‐AKT/AKT expression in mouse kidneys from each group. Data are presented as means ± SEM. Data are representative of three independent experiments, *P < 0.05, **P < 0.01, ***P < 0.001 vs. ADPKD, ns, not significant, Student's *t*‐test.

The above mentioned results demonstrated that PF‐06409577 could inhibit renal cyst formation and development in both *in vitro* and *in vivo* models. Next, we examined the underlying molecular mechanisms for its inhibitory effect. Incubation of MDCK cells with PF‐06409577 promoted a concentration‐dependent increase in AMPK phosphorylation, while no significant effect on total AMPK expression (Fig. 4A,C). This led to the inhibition of downstream mTOR signaling pathways (Fig. 4A). As evidence, p‐p70S6K/p70S6K and p‐4EBP1/4EBP1 were significantly down‐regulated (Fig. 4D,E). Similarly, treatment of ADPKD mice with 10 mg·kg^−1^ PF‐06409577 (Fig. 4B, G–I) resulted in significant AMPK phosphorylation (*P* = 0.0047, Student's *t* test, *n* = 3), down‐regulated p70S6K phosphorylation (*P* = 0.0273, Student's *t* test, *n* = 3) and 4EBP1 phosphorylation (*P* = 0.0009, Student's *t* test, *n* = 3). These data proved PF‐06409577 was capable of modulating epithelial cell proliferation through inhibiting the mTOR signaling pathway. Meanwhile, we found that AKT phosphorylation was not significantly affected by PF‐06409577 treatment both *in vitro* (Fig. 4A,F) and *in vivo* (Fig. 4B,J). This indicated that PF‐06409577 regulated the mTOR signaling pathway via AMPK, an AKT‐independent mechanism, rather than the traditional AKT‐dependent manner.

### PF‐06409577 inhibited CFTR‐dependent short‐circuit current in MDCK cells

**Fig. 5:**
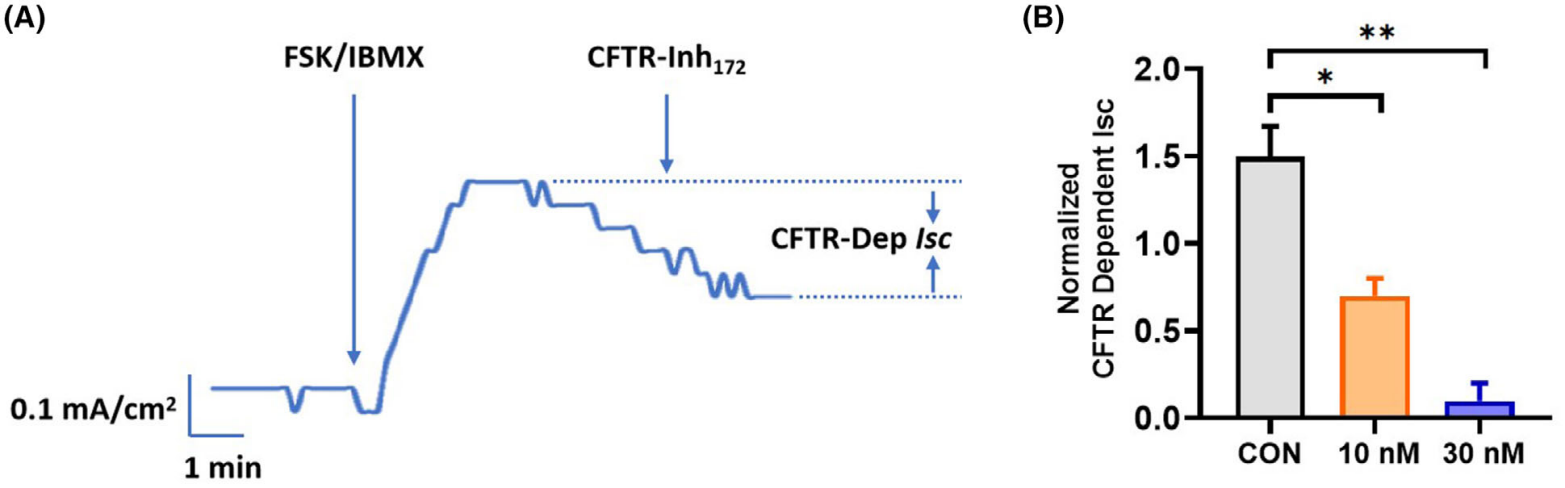
PF‐06409577 inhibited CFTR‐dependent short‐circuit current (*I*_*SC*_) in MDCK cells. (A) Representative *I*_*SC*_ trace in MDCK cells. (B) Quantification of the normalized CFTR‐dependent *I*_*SC*_ in MDCK cells pre‐treated with vehicle or different concertation of PF‐06409577 (10 or 30 nm). Data are presented as means ± SEM. *n* = 3, *P < 0.05, **P < 0.01 vs. Control (CON), one‐way ANOVA followed by Tukey's analysis.

Because CFTR is responsible for the fluid secretion into the cyst lumen, we next examined the inhibitory effect of PF‐06409577 on the CFTR function. MDCK cells were pre‐treated with 0, 10 or 30 nm PF‐06409577 for 1 h. The CFTR‐dependent short‐circuit current (*I*_*SC*_) in MDCK cells was recorded by stimulating MDCK cells with IBMX and FSK. Subsequently, MDCK cells were treated with the specific CFTR inhibitor CFTR_Inh_‐172 [7]. Typical trace of *I*_*SC*_ change was shown in Fig. 5A. Following IBMX/forskolin treatment, MDCK cells showed an early peak in *I*_*SC*_. This current was sensitive to inhibition by CFTR_Inh_‐172. The normalized CFTR‐dependent *I*_*SC*_ of each group was shown in Fig. 5B. The results demonstrated that PF‐06409577 reduced the short‐circuit current in MDCK cells. The effect was concentration‐dependent (*P* = 0.0161 at 10 nm; *P* = 0.0022 at 30 nm; one‐way ANOVA with Tukey's analysis relative to vehicle‐treated control; *n* = 3 for each condition). These results confirmed that PF‐06409577 had a significant inhibitory effect on CFTR function, which was responsible for its inhibition of renal cyst expansion.

## Discussion

PF‐06409577 is a selective AMPK activator. By acting through AMPK, this compound has shown efficacy in osteosarcoma [19], diabetic nephropathy [12], non‐alcoholic fatty liver disease (NAFLD) and non‐alcoholic steatohepatitis (NASH) [20], to name a few. In this study, we demonstrated that PF‐06409577 was efficacious in inhibiting renal cyst development in both cellular and embryonic kidney cyst models. By testing PF‐06409577 in a rapidly progressive ADPKD mouse model, we found the potential therapeutic effects of PF‐06409577 to retard renal cyst development in ADPKD *in vivo*. Further exploitation of the underlying molecular mechanisms revealed that PF‐06409577 stimulated AMPK phosphorylation, leading to concurrent inhibition of the mTOR pathway and CFTR chloride channel function. These collectively delayed the progression of ADPKD by inhibiting renal epithelial cell proliferation and cystic fluid secretion.

Aside from concurrent inhibition of mTOR and CFTR, numerous therapies targeting one of the key processes in the pathogenies of ADPKD are in development or in clinical trials [21, 22, 23, 24]. However, none of these strategies has successfully launched to the market. For instance, clinical trials of sirolimus, an mTOR inhibitor efficacious in preclinical trials, showed no clinical benefit, mostly due to its toxicity prevented adequate dosing to delay the disease progression in human ADPKD [22]. In comparison, PF‐06409577 offers the significant advantage of blocking two processes by acting through AMPK and exerts a cumulative inhibitory effect even at a relatively low dose. Moreover, the pathology of ADPKD *per se* is complex and involves the malfunction of different signaling pathways [3, 4]. Thus, concurrently inhibiting mTOR and CFTR may lead to synergistic therapeutic effects for ADPKD than using inhibitors of a single signaling pathway.

mTOR is a critical regulator of cell proliferation and cyst expansion in ADPKD [25]. The prototypical regulatory mechanism of mTOR is through activation of the canonical PI3K/AKT/mTOR signaling pathway [26]. For example, in diabetic kidney disease, Insulin or VEGF signaling‐induced AKT phosphorylation and protected kidneys via anti‐apoptosis and anti‐inflammatory effects in podocytes [27, 28, 29]. However, mTOR converges input from multiple signaling pathways, which are regulated not only by the AKT‐dependent manner but also by AKT‐independent manners. Herein, PF‐06409577 significantly down‐regulated the mTOR signaling pathway, yet without affecting AKT phosphorylation in ADPKD models. Similarly, Inoki et al. [10] reported that the activation of AMPK under energy starvation conditions directly phosphorylated TSC2 and enhanced its activity, thereby inhibiting downstream mTOR signaling pathway. Previous studies suggested that AKT phosphorylation was closely linked to ADPKD progression [25]. Thus, the AKT‐independent mTOR inhibition by PF‐06409577 might be advantageous for the treatment of ADPKD.

Similar to our finding, multiple preclinical studies also highlighted AMPK as a potential therapeutic target for ADPKD. A previous study by Takiar et al. [7] highlighted the therapeutic effect of metformin to retard disease progression in ADPKD. Pastor‐Soler et al. [30] tested the effectiveness of metformin as a therapy for ADPKD in a slowly progressing ADPKD model (i.e., *Pkd1* ^*RC/RC*^ mouse) and demonstrated that metformin improved renal function and reduced kidney injury in ADPKD. In another study, Lian et al. proved the combination of metformin with 2‐deoxyglucose efficaciously inhibited glycolysis [31] in a miniature pig model of ADPKD, hence significantly inhibiting the progression of renal cysts and improving renal function [32]. Notably, different from the action of PF‐06409577, metformin represents an indirect AMPK activator that disturbs mitochondrial complex I of the respiratory chain and suppresses ATP production [33, 34], which leads to increased intracellular AMP level and thus AMPK activation [33, 34, 35, 36]. Metformin also inhibited mitochondrial glycerol 3‐phosphate dehydrogenase, inhibiting NF‐κB, and activating mitophagy through Pink1 and Parkin [37]. Such mechanism of action is ubiquitous throughout the body, which, as a result, extends beyond the therapeutic role of metformin in ADPKD with potential side effects. By contrast, PF‐06409577 offers spatio‐temporal selectivity by directly activating α1β1γ1 and α2β1γ1 AMPK isoforms, two isoforms highly expressed in the kidney [12]. In addition, PF‐06409577 is highly potent in inhibiting the two AMPK isoforms with EC_50_ values of 7.0 and 6.8 nm [12, 19]. This offers a significant advantage over metformin for ADPKD treatment since the inhibitory dosage of metformin (1 mm for *in vitro* renal cyst models and 300 mg·kg^−1^·day^−1^ for the *Pkd1* ^*flox/flox*^ *;Ksp‐Cre* mouse model [7]) greatly exceeds the maximum PF‐06409577 dosage in our experiments (10 μm *in vitro* and 10 mg·kg^−1^·day^−1^ *in vivo*, respectively). At such high concentration, metformin has been shown the possibility to induce severe lactic acidosis [35]. Given its better selectivity and higher potency, PF‐06409577 might be repurposed as a candidate drug for ADPKD treatment.

Herein we focused on the anti‐cystogenesis activity of PF‐06409577 through AMPK activation. However, AMPK is the principal energy sensor ubiquitously expressed in eukaryotic cells [38, 39]. There are a number of studies suggests that metformin involves several side effects such as lactic acidosis [40, 41]. Accordingly, it is important to consider whether PF‐06409577 shares metformin's risk of developing lactic acidosis. As mentioned above, metformin acts indirectly by inhibiting mitochondrial complex I of the respiratory chain which is likely to explain the life‐threatening side effect of lactic acidosis [42]. It is therefore plausible that PF‐06409577 will be devoid of lactic acidosis by directly acting on AMPK without influencing mitochondrial Complex I. The long‐term adverse effect of chronic drug treatment is another issue to consider. It has been reported that vitamin B12 deficiency happened in 30% of patients with chronic metformin use [43, 44]. Admittedly, our study has some limitations due to the lack of a slowly progressing ADPKD model. Herein, we merely examined the efficacy of PF‐06409577 in a rapidly progressive ADPKD mouse model, and yet ADPKD patients need life‐long medical care [45]. Previous research demonstrated that AMPK activators were efficacious in several slowly progressing models of ADPKD. For example, the beneficial effects of metformin have been reported in *Pkd1* ^*RC/RC*^ mouse model [30] and ADPKD miniature pigs [32]. Similarly, Leonhard et al. [46] reported the beneficial effects of salsalate, a direct AMPK activator, in a tamoxifen‐induced conditional *Pkd1* knock‐out mouse model. Based on these results, we expect that PF‐06409577 may display a similar therapeutic effect for long‐term treatment for ADPKD. Investigating the long‐term outcomes of PF‐06409577 treatment, including both the therapeutic advantages and potential side effects, in ADPKD models will be an interesting area for future investigations.

In summary, PF‐06409577 effectively attenuated renal cyst development *in in vitro* and *in vivo* models of ADPKD. This effect was mediated by concurrently suppressing the mTOR pathway and CFTR function. Therefore, PF‐06409577 represents a potential drug candidate for the treatment of ADPKD.

## Conflict of interest

The authors declare no conflict of interest.

## Author contributions

DG and JW conceived and supervised the study; DG, LS and HY designed experiments; LS, HY, HZ and RW performed experiments; YR and KF provided new tools and reagents; QF, HL and LY developed new software and performed simulation studies; DG, LS and HY analyzed data; LS wrote the manuscript and DG and JW made manuscript revisions.

## Data Availability

The data that support the findings of this study are available from the corresponding author [guo@xzhmu.edu.cn] upon reasonable request.

## References

[1] Wilson PD . Polycystic kidney disease. N Engl J Med. 2004;350(2):151–64. 14711914 10.1056/NEJMra022161

[2] Mochizuki T , Wu G , Hayashi T , Xenophontos SL , Veldhuisen B , Saris JJ , et al. PKD2, a gene for polycystic kidney disease that encodes an integral membrane protein. Science. 1996;272(5266):1339–42. 8650545 10.1126/science.272.5266.1339

[3] Bergmann C , Guay‐Woodford LM , Harris PC , Horie S , Peters DJ , Torres VE . Polycystic kidney disease. Nat Rev Dis Primers. 2018;4(1):50. 30523303 10.1038/s41572-018-0047-y PMC6592047

[4] Cornec‐Le GE , Alam A , Perrone RD . Autosomal dominant polycystic kidney disease. Lancet. 2019;393(10174):919–35. 30819518 10.1016/S0140-6736(18)32782-X

[5] Menezes LF , Germino GG . The pathobiology of polycystic kidney disease from a metabolic viewpoint. Nat Rev Nephrol. 2019;15(12):735–49. 31488901 10.1038/s41581-019-0183-y

[6] Pinto CS , Raman A , Reif GA , Magenheimer BS , White C , Calvet JP , et al. Phosphodiesterase isoform regulation of cell proliferation and fluid secretion in autosomal dominant polycystic kidney disease. J Am Soc Nephrol. 2016;27(4):1124–34. 26289612 10.1681/ASN.2015010047 PMC4814181

[7] Takiar V , Nishio S , Seo‐Mayer P , King JD Jr , Li H , Zhang L , et al. Activating AMP‐activated protein kinase (AMPK) slows renal cystogenesis. Proc Natl Acad Sci USA. 2011;108(6):2462–7. 21262823 10.1073/pnas.1011498108 PMC3038735

[8] Cabrita I , Kraus A , Scholz JK , Skoczynski K , Schreiber R , Kunzelmann K , et al. Cyst growth in ADPKD is prevented by pharmacological and genetic inhibition of TMEM16A in vivo. Nat Commun. 2020;11(1):4320. 32859916 10.1038/s41467-020-18104-5 PMC7455562

[9] Davidow CJ , Maser RL , Rome LA , Calvet JP , Grantham JJ . The cystic fibrosis transmembrane conductance regulator mediates transepithelial fluid secretion by human autosomal dominant polycystic kidney disease epithelium in vitro. Kidney Int. 1996;50(1):208–18. 8807590 10.1038/ki.1996.304

[10] Inoki K , Zhu T , Guan KL . TSC2 mediates cellular energy response to control cell growth and survival. Cell. 2003;115(5):577–90. 14651849 10.1016/s0092-8674(03)00929-2

[11] Hallows KR , Raghuram V , Kemp BE , Witters LA , Foskett JK . Inhibition of cystic fibrosis transmembrane conductance regulator by novel interaction with the metabolic sensor AMP‐activated protein kinase. J Clin Invest. 2000;105(12):1711–21. 10862786 10.1172/JCI9622 PMC378514

[12] Cameron KO , Kung DW , Kalgutkar AS , Kurumbail RG , Miller R , Salatto CT , et al. Discovery and preclinical characterization of 6‐Chloro‐5‐[4‐(1‐hydroxycyclobutyl)phenyl]‐1H‐indole‐3‐carboxylic acid (PF‐06409577), a direct activator of adenosine monophosphate‐activated protein kinase (AMPK), for the potential treatment of diabetic nephropathy. J Med Chem. 2016;59(17):8068–81. 27490827 10.1021/acs.jmedchem.6b00866

[13] Su L , Liu L , Jia Y , Lei L , Liu J , Zhu S , et al. Ganoderma triterpenes retard renal cyst development by downregulating Ras/MAPK signaling and promoting cell differentiation. Kidney Int. 2017;92(6):1404–18. 28709639 10.1016/j.kint.2017.04.013

[14] Zhang H , Yan W , Sun Y , Yuan H , Su L , Cao X , et al. Long residence time at the vasopressin V_2_ receptor translates into superior inhibitory effects in ex vivo and in vivo models of autosomal dominant polycystic kidney disease. J Med Chem. 2022;65(11):7717–28. 35363466 10.1021/acs.jmedchem.2c00011

[15] Cao X , Wang P , Yuan H , Zhang H , He Y , Fu K , et al. Benzodiazepine derivatives as potent vasopressin V_2_ receptor antagonists for the treatment of autosomal dominant kidney disease. J Med Chem. 2022. 10.1021/acs.jmedchem.2c00567 35579344

[16] He J , Zhou H , Meng J , Zhang S , Li X , Wang S , et al. Cardamonin retards progression of autosomal dominant polycystic kidney disease via inhibiting renal cyst growth and interstitial fibrosis. Pharmacol Res. 2020;155:104751. 32151678 10.1016/j.phrs.2020.104751

[17] Wang W , Geng X , Lei L , Jia Y , Li Y , Zhou H , et al. Aquaporin‐3 deficiency slows cyst enlargement in experimental mouse models of autosomal dominant polycystic kidney disease. FASEB J. 2019;33(5):6185–96. 30768374 10.1096/fj.201801338RRR PMC6463927

[18] Shibazaki S , Yu Z , Nishio S , Tian X , Thomson RB , Mitobe M , et al. Cyst formation and activation of the extracellular regulated kinase pathway after kidney specific inactivation of Pkd1. Hum Mol Genet. 2008;17(11):1505–16. 18263604 10.1093/hmg/ddn039 PMC2902289

[19] Zhu Y , Zhang X , Wu Q , Yu C , Liu Y , Zhang Y . PF‐06409577 activates AMPK signaling and inhibits osteosarcoma cell growth. Front Oncol. 2021;11:659181. 34336655 10.3389/fonc.2021.659181 PMC8316637

[20] Esquejo RM , Salatto CT , Delmore J , Albuquerque B , Reyes A , Shi Y , et al. Activation of liver AMPK with PF‐06409577 corrects NAFLD and lowers cholesterol in rodent and primate preclinical models. EBioMedicine. 2018;31:122–32. 29673898 10.1016/j.ebiom.2018.04.009 PMC6014578

[21] Chebib FT , Torres VE . Recent advances in the Management of Autosomal Dominant Polycystic Kidney Disease. Clin J Am Soc Nephrol. 2018;13(11):1765–76. 30049849 10.2215/CJN.03960318 PMC6237066

[22] Serra AL , Poster D , Kistler AD , Krauer F , Raina S , Young J , et al. Sirolimus and kidney growth in autosomal dominant polycystic kidney disease. N Engl J Med. 2010;363(9):820–9. 20581391 10.1056/NEJMoa0907419

[23] Tesar V , Ciechanowski K , Pei Y , Barash I , Shannon M , Li R , et al. Bosutinib versus placebo for autosomal dominant polycystic kidney disease. J Am Soc Nephrol. 2017;28(11):3404–13. 28838955 10.1681/ASN.2016111232 PMC5661280

[24] Caroli A , Perico N , Perna A , Antiga L , Brambilla P , Pisani A , et al. Effect of longacting somatostatin analogue on kidney and cyst growth in autosomal dominant polycystic kidney disease (ALADIN): a randomised, placebo‐controlled, multicentre trial. Lancet. 2013;382(9903):1485–95. 23972263 10.1016/S0140-6736(13)61407-5

[25] Chapin HC , Caplan MJ . The cell biology of polycystic kidney disease. J Cell Biol. 2010;4(191):701–10. 10.1083/jcb.201006173 PMC2983067 21079243

[26] Margaria JP , Campa CC , De‐Santis MC , Hirsch E , Franco I . The PI3K/Akt/mTOR pathway in polycystic kidney disease: a complex interaction with polycystins and primary cilium. Cell Signal. 2020;66:109468. 31715259 10.1016/j.cellsig.2019.109468

[27] Mima A , Ohshiro Y , Kitada M , Matsumoto M , Geraldes P , Li C , et al. Glomerular‐specific protein kinase C‐beta‐induced insulin receptor substrate‐1 dysfunction and insulin resistance in rat models of diabetes and obesity. Kidney Int. 2011;79(8):883–96. 21228767 10.1038/ki.2010.526 PMC3612886

[28] Mima A , Kitada M , Geraldes P , Li Q , Matsumoto M , Mizutani K , et al. Glomerular VEGF resistance induced by PKCdelta/SHP‐1 activation and contribution to diabetic nephropathy. FASEB J. 2012;7(26):2963–74. 10.1096/fj.11-202994 PMC3382088 22499584

[29] Mima A , Yasuzawa T , Nakamura T , Ueshima S . Linagliptin affects IRS1/Akt signaling and prevents high glucose‐induced apoptosis in podocytes. Sci Rep. 2020;1(10):5775. 10.1038/s41598-020-62579-7 PMC7113296 32238837

[30] Pastor‐Soler NM , Li H , Pham J , Rivera D , Ho P , Mancino V , et al. Metformin improves relevant disease parameters in an autosomal dominant polycystic kidney disease mouse model. Am J Physiol Renal Physiol. 2022;1(322):F27–41. 10.1152/ajprenal.00298.2021 34806449

[31] Brown J . Effects of 2‐deoxyglucose on carbohydrate metablism: review of the literature and studies in the rat. Metabolism. 1962;11:1098–112. 13873661

[32] Lian X , Wu X , Li Z , Zhang Y , Song K , Cai G , et al. The combination of metformin and 2‐deoxyglucose significantly inhibits cyst formation in miniature pigs with polycystic kidney disease. Br J Pharmacol. 2019;5(176):711–24. 10.1111/bph.14558 PMC6365356 30515768

[33] Owen MR , Doran E , Halestrap AP . Evidence that metformin exerts its anti‐diabetic effects through inhibition of complex 1 of the mitochondrial respiratory chain. Biochem J. 2000;348(Pt 3):607–14. 10839993 PMC1221104

[34] El‐Mir MY , Nogueira V , Fontaine E , Avéret N , Rigoulet M , Leverve X . Dimethylbiguanide inhibits cell respiration via an indirect effect targeted on the respiratory chain complex I. J Biol Chem. 2000;275(1):223–8. 10617608 10.1074/jbc.275.1.223

[35] Foretz M , Hébrard S , Leclerc J , Zarrinpashneh E , Soty M , Mithieux G , et al. Metformin inhibits hepatic gluconeogenesis in mice independently of the LKB1/AMPK pathway via a decrease in hepatic energy state. J Clin Invest. 2010;120(7):2355–69. 20577053 10.1172/JCI40671 PMC2898585

[36] Argaud D , Roth H , Wiernsperger N , Leverve XM . Metformin decreases gluconeogenesis by enhancing the pyruvate kinase flux in isolated rat hepatocytes. Eur J Biochem. 1993;213(3):1341–8. 8504825 10.1111/j.1432-1033.1993.tb17886.x

[37] Mima A . Mitochondria‐targeted drugs for diabetic kidney disease. Heliyon. 2022;2(8):e08878. 10.1016/j.heliyon.2022.e08878 PMC8899696 35265754

[38] Hardie DG . AMP‐activated/SNF1 protein kinases: conserved guardians of cellular energy. Nat Rev Mol Cell Biol. 2007;8(10):774–85. 17712357 10.1038/nrm2249

[39] Hardie DG , Schaffer BE , Brunet A . AMPK: an energy‐sensing pathway with multiple inputs and outputs. Trends Cell Biol. 2016;26(3):190–201. 26616193 10.1016/j.tcb.2015.10.013 PMC5881568

[40] Flory J , Lipska K . Metformin in 2019. JAMA. 2019;321(19):1926–7. 31009043 10.1001/jama.2019.3805 PMC7552083

[41] Crowley MJ , Diamantidis CJ , McDuffie JR , Cameron CB , Stanifer JW , Mock CK , et al. Clinical outcomes of metformin use in populations with chronic kidney disease, congestive heart failure, or chronic liver disease: a systematic review. Ann Intern Med. 2017;166(3):191–200. 28055049 10.7326/M16-1901 PMC5293600

[42] Hardie DG . Role of AMP‐activated protein kinase in the metabolic syndrome and in heart disease. FEBS Lett. 2008;582(1):81–9. 18022388 10.1016/j.febslet.2007.11.018

[43] Thomas I , Gregg B . Metformin; a review of its history and future: from lilac to longevity. Pediatr Diabetes. 2017;18(1):10–6. 28052534 10.1111/pedi.12473

[44] Hameed M , Khan K , Salman S , Mehmood N . Dose comparison and side effect profile of metformin extended release versus metformin immediate release. J Ayub Med Coll Abbottabad. 2017;29(2):225–9. 28718236

[45] Brosnahan GM , Abebe KZ , Rahbari‐Oskoui FF , Patterson CG , Bae KT , Schrier RW , et al. Effect of statin therapy on the progression of autosomal dominant polycystic kidney disease. A secondary analysis of the HALT PKD trials. Curr Hypertens Rev. 2017;13(2):109–20. 28460625 10.2174/1573402113666170427142815 PMC5688015

[46] Leonhard WN , Song X , Kanhai AA , Iliuta IA , Bozovic A , Steinberg GR , et al. Salsalate, but not metformin or canagliflozin, slows kidney cyst growth in an adult‐onset mouse model of polycystic kidney disease. EBioMedicine. 2019;47:436–45. 31473186 10.1016/j.ebiom.2019.08.041 PMC6796518

